# Association of Hepatitis B Virus Covalently Closed Circular DNA and Human APOBEC3B in Hepatitis B Virus-Related Hepatocellular Carcinoma

**DOI:** 10.1371/journal.pone.0157708

**Published:** 2016-06-16

**Authors:** Xuan Luo, Yao Huang, Yanmeng Chen, Zeng Tu, Jieli Hu, John E. Tavis, Ailong Huang, Yuan Hu

**Affiliations:** 1 Key Laboratory of Molecular Biology on Infectious Diseases, Ministry of Education, Institute for Viral Hepatitis, Department of Infectious Diseases, Second Affiliated Hospital, Chongqing Medical University, Chongqing, People’s Republic of China; 2 Department of Microbiology, College of Basic Medical Sciences, Chongqing Medical University, Chongqing, People’s Republic of China; 3 Department of Molecular Microbiology and Immunology, Saint Louis University Liver Center, Saint Louis University School of Medicine, Saint Louis, Missouri, United States of America; 4 Collaborative Innovation Center for Diagnosis and Treatment of Infectious Diseases, Zhejiang University, Hangzhou, People’s Republic of China; Drexel University College of Medicine, UNITED STATES

## Abstract

Chronic Hepatitis B Virus (HBV) infections can progresses to liver cirrhosis and hepatocellular carcinoma (HCC). The HBV covalently-closed circular DNA cccDNA is a key to HBV persistence, and its degradation can be induced by the cellular deaminase APOBEC3. This study aimed to measure the distribution of intrahepatic cccDNA levels and evaluate the association between levels of cccDNA and APOBEC3 in HCC patients. Among 49 HCC patients, 35 matched cancerous and contiguous noncancerous liver tissues had detectable cccDNA, and the median intrahepatic cccDNA in the cancerous tissues (CT) was significantly lower than in the contiguous noncancerous tissues (CNCT) (p = 0.0033). RCA (rolling circle amplification), followed by 3D-PCR identified positive amplification in 27 matched HCC patients. Sequence analysis indicated G to A mutations accumulated to higher levels in CT samples compared to CNCT samples, and the dinucleotide context showed preferred editing in the GpA context. Among 7 APOBEC3 genes, APOBEC3B was the only one up-regulated in cancerous tissues both at the transcriptional and protein levels (p < 0.05). This implies APOBEC3B may contribute to cccDNA editing and subsequent degradation in cancerous tissues.

## Introduction

Hepatitis B virus (HBV) infection is a major health problem world-wide. Up to 350 million people have chronic infection and are at high risk of progressing to liver cirrhosis and hepatocellular carcinoma (HCC) [1,2].

HBV is a partially double-stranded DNA virus that replicates by reverse transcription which occurs within viral core particles in the cytoplasm [3,4]. Newly synthesized viral genomes can either be secreted as virions, or they can be transported into the nucleus where the relaxed circular DNA (RC DNA) is converted to covalently-closed circular DNA (cccDNA). The nuclear cccDNA accumulates to about 1 to 50 copies per hepatocyte in animal models as fairly stable mini-chromosomes [5,6]. Despite this low accumulation level, the cccDNA is key to HBV persistence because it is the template for the all HBV mRNAs, including the pregenomic RNA (pgRNA) that is converted to DNA during reverse transcription [7].

Previously, extensive studies have shown that HBV cccDNA persists throughout the various clinical phases of chronic hepatitis [8,9]. Even in patients with occult HBV infection who are negative for hepatitis B surface antigen (HBsAg) but have detectable HBV DNA in sera or liver tissues, cccDNA is still present [10,11], although at low levels. Clinical reports stressed the importance of measuring levels of cccDNA due to its key position in the viral replication cycle [8]. In addition, intrahepatic cccDNA levels vary between the immune-tolerant phase and immune-clearance phage of chronic HBV infection [12]. This implies that host factors such as hepatocyte turnover and inflammatory cytokines may affect cccDNA levels [7,13].

The apolipoprotein B mRNA-editing catalytic polypeptide 3 (APOBEC3) family is comprised of seven DNA cytidine deaminases (APOBEC3A, B, C, D, F, G and H) in humans. These proteins can bind to target DNA and convert cytosine to uracil [14–16]. As innate antiviral factors, APOBEC3 proteins can edit HBV genome and reduce HBV replication *in vitro* and *in vivo* [17–19]. HBV DNA carrying C to U modifications can subsequently be degraded, or alternatively, the large number of G to A mutations that accumulate in the plus strand DNA during reverse transcription could render it non-infectious. Recently, Kitamura et al demonstrated that Duck HBV cccDNA accumulated G to A hypermutations that were induced by APOBEC3G, and the lesions were repaired by the uracil-DNA glycosylase (UNG)- mediated base-excision repair (BER) pathway [20]. Another intriguing study reported IFN -α and lymphotoxin-β-receptor could up-regulate APOBEC3A and 3B, respectively, leading to cytidine deamination- dependent cccDNA degradation [21]. These two reports imply that APOBEC3 could target double-strand cccDNA and decrease its levels in the nucleus.

Considering important role of cccDNA in chronic hepatitis B infection (CHB), conducting clinical research to verify this association of cccDNA levels and APOBEC3 proteins is important. Therefore, we measured the levels of cccDNA in HCC tissues and then examined whether APOBEC3B may be a candidate host factor for cccDNA editing that could decrease levels cccDNA in HCC. These studies employed cccDNA quantification, analysis cccDNA mutation patterns, evaluation of APOBEC3B expression levels in paired CT and CNCT samples of HCC patients.

## Materials and Methods

### Patients

Matched cancerous and contiguous noncancerous liver tissues were obtained from 49 HCC patients who underwent surgical resection at the Chongqing Medical University First and Second Hospitals. All patients were ethnic Han Chinese. HBsAg-positive carriers (*n* = 45) and HBsAg-negative/ hepatitis B core antibody (HBcAb) -positive patients (*n* = 4) were enrolled. Diagnosis of HCC for these samples was confirmed by histopathology. Hepatitis C virus (HCV) or Human Immunodeficiency Virus (HIV) infected patients defined by anti-HCV or anti -HIV positivity were excluded as APOBEC3 expression could be affected by coinfection [22]. Serum HBsAg, anti-HBs, HBeAg, anti-HBe, anti-HBc were measured by ELISA (Kehua, Shanghai, China) according to the manufacturer’s instruction. Tissue samples were rapidly frozen in liquid nitrogen and stored at –80°C until use.

### Plasmids employed

The expression vector for hemagglutinin (HA)-tagged APOBEC3B was constructed by Genecopoeia Company. The human APOBEC3B (NM_004900.4) was cloned with a C-terminal HA-tag in pReceiver vector.

pCH9/3091 is an HBV expression vector contains 1.1 copies of HBV (genotype D) genome. It was obtained from Dr. Lin Lan (The Third Military Medical University, Chongqing, China).The YMHA derivative of this plasmid was made by introducing YMDD to YMHA mutations into the HBV P gene at this key active site motif.

### Quantitation of intrahepatic HBV cccDNA and HBV total DNA

Intrahepatic HBV total DNA was extracted from 20mg of liver tissues using QIAamp

DNA Mini Kit (QIAGEN GmbH, Hilden, Germany) according to the manufacturer’s instructions, then the extracted DNAs were treated with DNA-safe ATP dependent enzyme (PSAD) (Epicentre, Madison, WI) to digest HBV linear double-strands (dsl) and single-strand (ss) DNA in preparation for cccDNA detection and amplification.

We then performed real-time PCR with a TagMan probe to quantify intrahepatic HBV cccDNA and total HBV DNA. For the HBV cccDNA, a pair of cccDNA-selective primers and a probe were selected that targets the gap region between the two direct repeat regions (DR1 and DR2) of the viral genome [8]. Intrahepatic HBV cccDNA and total DNA levels were normalized by the amount of β-Globin DNA in the samples. Cell numbers were calculated based on an estimation of 6.667 pg/hgDNA per cell [9]. The primers and probe for HBV total DNA and β-Globin are shown in S1 Table.

### Mutation analysis of cccDNA by RCA and 3D-PCR

HBV cccDNA was preferentially amplified relative to the other HBV DNA forms by rolling circle amplification (RCA) [23]. Briefly, cccDNA from liver tissue was amplified by phi-29 DNA polymerase mixed with HBV specific primers as described, then the concatemerized RCA product was amplified with full-length HBV genomic PCR.

The core region was amplified by employing 3D-PCR for mutational analysis of the cccDNA: Concatemerized PCR products from RCA reaction were used as templates for 3D-PCR. The amplification conditions were: 5 min (82–86°C), then 20 sec at 82–86°C; 30 sec at 51.5°C; and 50 sec at 72°C for 35 cycles, then at 10 min 72°C. The primers for full-length genomic PCR and 3D-PCR are in S1 Table.

### Quantitation of AP sites of cccDNA

To measure the AP sites in cccDNA-containing samples, 1μg of cccDNA-containing extracts was treated with apuricic/apyrimidinic endonuclease (APE1) (New England Biolabs, Ipswich, MA, USA) for 4h at 37°C, then the levels of cccDNA were measured before and after treatment by real time PCR with a cccDNA specific probe described above.

### Quantitation of APOBEC3 transcript profiles

Total RNA was isolated from the liver samples using Trizol reagent (Life Technologies, Carlsbad, CA, USA). 1 ug RNA was reverse transcribed into cDNA using GoScript^TM^ Reverse Transcription System (Promega, WI, USA), then the mRNA levels of the 7 APOBEC3 genes were measured by RT-qPCR using SYBRPremix Ex Taq (Takara Bio Inc, Ostu, Japan) as described earlier [24]. The measurement of each target mRNA level was calculated relative to constitutive housekeeping gene GAPDH using the 2^-ΔΔCT^ method.

### Immunoblotting analysis

Total protein was isolated from liver samples using RIPA (Beyotime, Beijing, China) with protease inhibitors cocktail (Roche, Indianapolis, USA). Expression level of APOBEC3A and APOBEC3B were analyzed by immunoblotting using primary antibodies specific for APOBEC3A and APOBEC3B (sc-130688, sc-130955; Santa Cruz Biotechnology, Dallas, USA), followed by secondary peroxidase-conjugated antibodies (ZSGB-BIO, Beijing, China) and chemiluminescence (Millipore Corporation, Billerica, USA).

### Isolation of viral DNA and Southern blot

For RCA control experiment, HBV cccDNA extraction from HepAD38 cells employed a modified Hirt procedure [25]. HBV DNA from core particles were isolated by mild detergent lysis of the cells and sedimentation through sucrose density gradient as described [26,27]. To detect HBV DNAs of RCA product, Southern blot was performed as reported [26] with minor modifications by using the DIG high prime DNA labeling and detection starter kit (Roche Diagnostics GmbH, Mannheim, German).

For APOBEC3B edit RNA experiments, Huh7.0 cells were maintained in Dulbecco’s modified Eagle medium with 10% fetal bovine serum. All transfections were performed using Mirus LT1 transfection reagent (Mirus Bio, Madison,WI, USA). Total RNA in the cell lysates was isolated using Trizol reagent (Life Technologies, Carlsbad, CA, USA). After digesting DNA in the samples with DNase I (Takara Bio Inc, Ostu, Japan), 1 μg of RNA was reverse transcribed into cDNA using GoScriptTM Reverse Transcription System (Promega, WI, USA), and analyzed by 3D-PCR.

### Statistical Analysis

All statistical analyses were performed by R language (The R Project for Statistical Computing). Comparisons of categorical variables (ratio of male: female, HBeAg, HBcAb) between groups were performed by Fisher exact test, while the Wilcoxon test was used for continuous variables (age, ALT AFP, serum DNA load). Differences in levels of HBV cccDNA or APOBEC3 in matched samples were analyzed by paired-Wilcoxon test, and correlations were by Pearson’s correlation analysis. The number of mutations was calculated by a perl script. A P value of < 0.05 was considered as statistically significant.

### Ethics

The study was approved by the Chinese Ethics Committee of Human Resources at the First and Second Hospitals of Chongqing Medical University. The participants provide their written informed consent to participate in this study. The Ethics Committee approved this consent procedure.

## Results

### Clinical Characteristics of the Patients

Demographic data for the 49 HCC patients are in Table 1. The average age was 51 years, and the average serum alanine aminotransferase (ALT) and HBV DNA levels were 87.65 IU/L and 9.37 x10^5^ copies/ml, respectively. The mean serum HBV DNA levels in the HBsAg-positive group were significantly higher than in the HBsAg-negative group (9.65 x10^5^ copy/ml versus 1.0 x 10^3^, p = 0.015).

**Table 1:** Clinical information of patients.

Characteristic	HBsAg-positive(n = 45)	HBsAg-negative(n = 4)	p value
**Male:Female ratio**	39:6	2:2	0.1195^b^
**HBeAg(+/-)**	13/32	1/3	0.629^b^
**HBcAb(+/-)**	33/2	4/0	0.3376^b^
**Mean age ± SD (years)**	49.64±12.90	69.5±9.57	0.0088^a^
**ALT(IU/L)**	92.51±152.28	34.75±18.52	0.433^a^
**AFP(ng/ml)**	1.94x 10^4^±8.54x 10^4^	42.1±67.86	0.212^a^
**Mean serum HBV DNA levels(copies/ml)**	9.65x 10^5^	1.0x 10^3^	0.015^a^

—: no detective data

a: Wilcox Test

b: Fisher exact test.

### Intrahepatic levels of HBV DNA and cccDNA in patients

We detected intrahepatic cccDNA and total HBV DNA in the matched CT and CNCT samples using qPCR. All 49 HCC patients, including both HBsAg-positive and -negative individuals had detectable total intrahepatic HBV DNA, and the majority of the samples had intrahepatic total HBV DNA levels above 1 copy/cell, 88% (43/49) in the CT group and 94% (46/49) in the CNCT groups.

**Fig 1:**
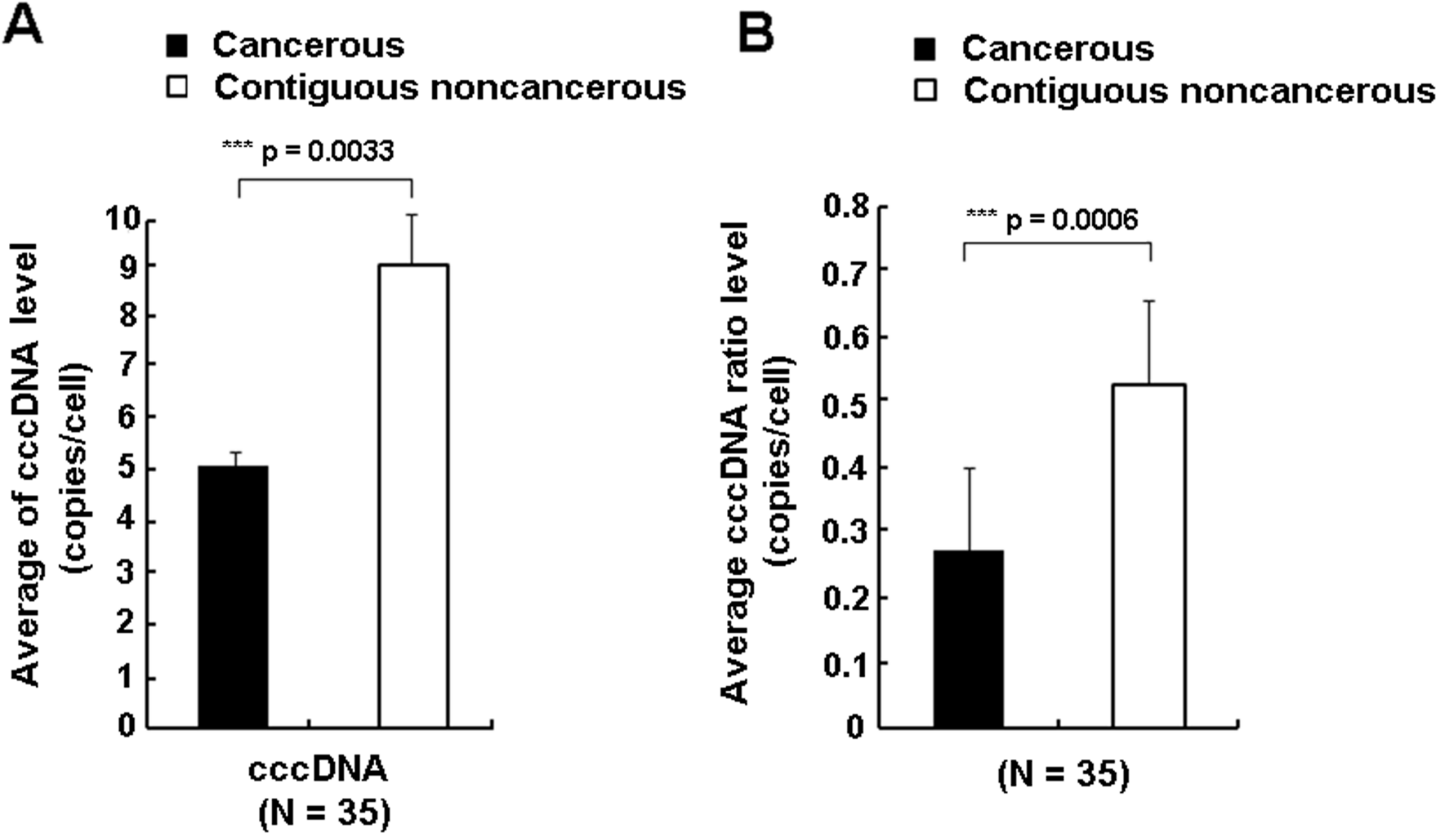
**Comparison of detectable intrahepatic cccDNA levels (A) and ratio of cccDNA/total HBV DNA (B) between the CT group (solid box) and matched CNCT group (empty box) for 35 HCC patients by paired-Wilcoxon test.** The levels of total DNA and cccDNA were normalized to copies/ cell.

Intrahepatic levels of cccDNA were relatively low in the 49 HCC patients. For the 4 HBsAg- negative but anti-HBc positive patients, cccDNA was detected in 2 CT and 3 CNCT samples, while among the 45 HBsAg-positive patients, cccDNA was detected in 33 CT and 45 CNCT samples. About 41% (20/49) of the CT group with cccDNA had values lower than 0.1copies/cell, compared to 12% (6/49) of the CNCT group. The mean intrahepatic cccDNA in the CT group was about 1.8-fold lower than in the CNCT group (5.20 copies/cell versus 9.35 copies/cell, p = 0.0033, Fig 1A), and the mean ratio of cccDNA/total HBV DNA in CT group was also significantly lower than in the contiguous CNCT group (0.27 vs 0.53, p = 0.0006, Fig 1B).

**Fig 2:**
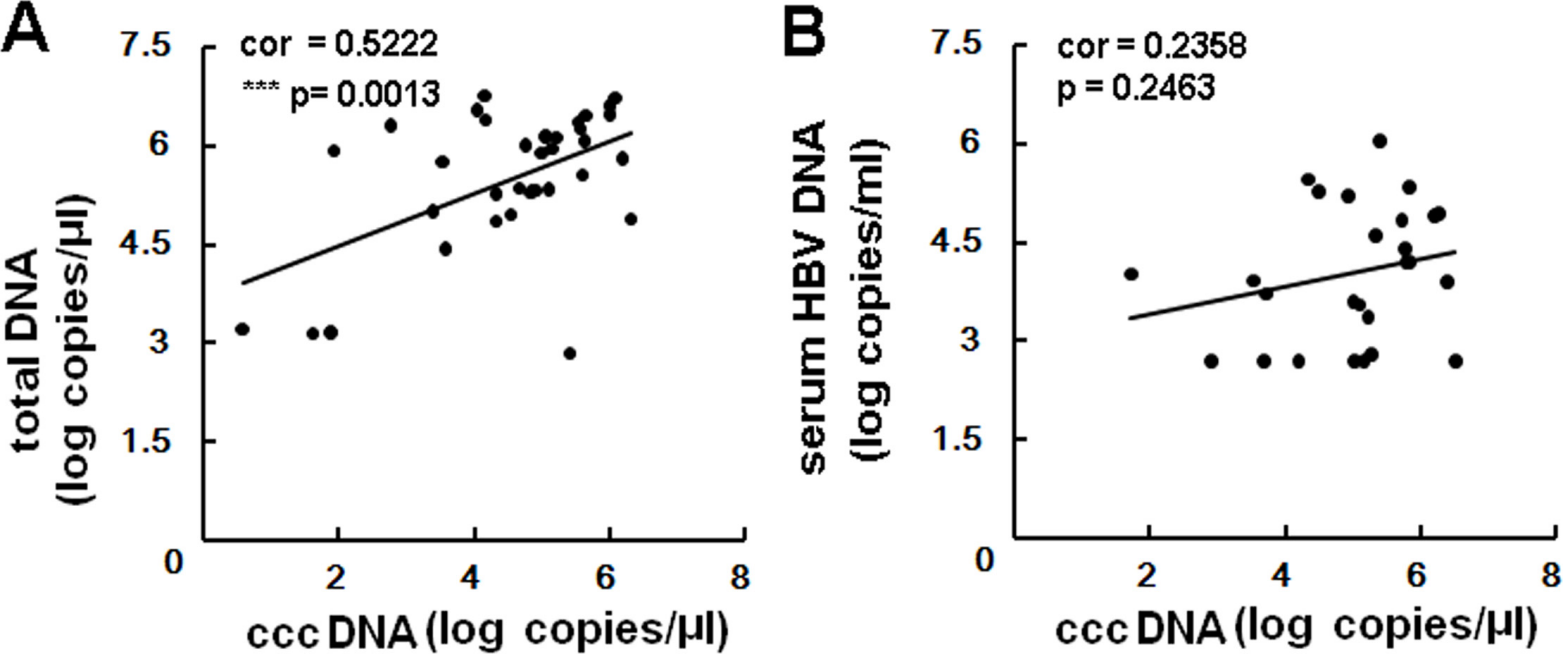
Correlation between intrahepatic HBV cccDNA and HBV total DNA or serum HBV DNA. A significant correlation was observed between total intrahepatic cccDNA levels and HBV DNA (panel **A**), but not for serum HBV DNA level (panel **B**) among 35 HCC patients with detectable cccDNA in paired samples.

We also analyzed the correlation between intrahepatic cccDNA levels and total HBV levels or serum HBV DNA levels. The values of intrahepatic cccDNA, total HBV DNA, serum DNA for this correlation analysis complied with standard normal distribution employing R language Shapiro-Wilk Normality Test (S2 Table). Among patients who had detectable cccDNA, a positive correlation was observed between intrahepatic cccDNA levels and HBV DNA (r2 = 0.5221, p = 0.0013), but not for serum HBV DNA levels (r2 = 0.2358, p = 0.2463) (Fig 2).

### Analysis of G to A mutations in HBV cccDNA in liver tissues

APOBEC3 proteins can inhibit HBV replication, especially APOBEC3A and APOBEC3B, by inducing HBV cccDNA degradation via a cytidine-deamination mechanism [21]. Therefore, we analyzed a possible correlation between APOBEC3 and HBV cccDNA levels in HCC patients by assessing the G to A mutation levels of cccDNA in the 49 HCC samples employed for cccDNA quantification.

RCA can be used not only for amplification of HBV cccDNA from liver biopsies [23], but also for preparation of randomly mutated plasmid libraries [28]. This let us to hypothesize RCA could be employed for cccDNA mutation analysis. The specificity of this method was confirmed with following validation assays. As shown in S1A Fig, only HBV cccDNA from HepAD38 or clinical liver tissues could be amplified by RCA, yielding a high molecular weight DNA product consisting of tandem repeats of HBV genome. As controls, HBV replication intermediates from cytoplasmic core particles or genomic liver DNA from HBV transgenic mice were both unable to be amplified by RCA due to lack of cccDNA. To recover sequence from cccDNA, the high molecular weight RCA products were amplified by HBV full-genomic PCR, yielding a clear band at 3.2kb (S1B Fig). Southern blot with HBV specific probe confirmed this band came from HBV (S1C Fig). Therefore, cccDNA sequence could be analyzed by combining RCA and PCR methods.

**Fig 3:**
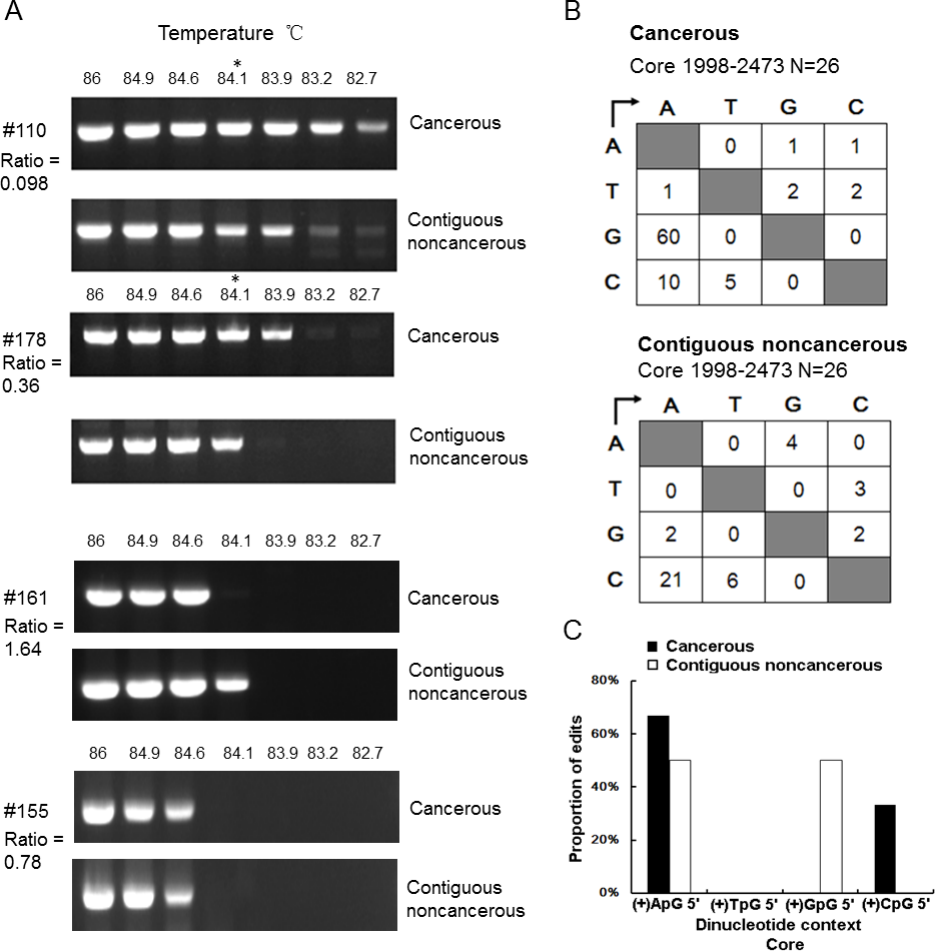
Mutational analysis of HBV cccDNA in HCC patients. **A.** Agarose gel analysis of 3D-PCR of cccDNA from CT and CNCT of samples from patients #110, #178, #161and #155. cccDNA was specifically amplified by RCA, then the concatemerized product was analysis by 3D-PCR using a denaturation temperature gradient of 86°C-82°C. **B**. Mutation matrices of cccDNA from CT and CNCT groups. DNA fragments amplified at 94°C (as reference wild type sequence) or 84.1°C in each group were cloned into T vectors, 13 clones were randomly selected, and the core region was sequenced. **C**. Dinucleotide editing profile of G to A mutations of plus-polarity strand DNA. Asterisks indicate the 3D-PCR products cloned and sequenced.

Next, to analyze G to A mutation levels in the paired 35 HCC samples with the detectable cccDNA, we employed RCA to amplify cccDNA specifically in these tissue samples first, then the RCA products were amplified by 3D-PCR as the 3D-PCR method can selectively amplify hypermutated DNA by lowering the denaturation temperature [29]. These 35 paired HCC samples were divided into 2 groups based on the ratio of cccDNA in the paired CT and CNCT samples (cccDNA value in CT / cccDNA value in CNCT) being either <1 or >1 (Table 2). Among these samples, 27 paired samples were amplified successfully in both tissue types, while 8 paired samples were not able to be amplified (Table 2, S2 Fig). For the 25 paired samples with ratio of cccDNA levels <1 (CT/CNCT), amplification of cccDNA from 16 paired CT group (such as #110, #178) employing a low denaturation temperature resulted in a relatively strong PCR signals compared with those from matched CNCT tissues (Fig 3A). For the 10 paired samples with ratio of cccDNA levels >1 (CT/CNCT), this 3D-PCR result was observed in 6 paired samples (Fig 3A, #161). Therefore, the positive results in 3D-PCR which were accordance with that ratio of cccDNA value between CT and CNCT are 62.85% (22/35, Table 2). In addition, there was no distinct difference in 3D-PCR patterns for 3 paired samples (Fig 3A, such as #155).

**Table 2:** 3D-PCR results of HCC patients.

Ratio of cccDNA (CT/CNCT)	Results of 3D-PCR			
	CT amplified at lower temperatures than CNCT	CT amplified at higher temperatures than CNCT	No difference in amplification at lower temperatures between CT and CNCT	Not amplifiable
**Ratio<1 (n = 25)**	16	2	2	5
**Ratio>1 (n = 10)**	0	6	1	3

We define positive change for the following results: the denaturation temperature is lower in CT sample than that in CNCT sample with cccDNA ratio <1 (example like #110, 178 in Fig 3) or the denaturation temperature is higher in CT sample than that in the CNCT sample for the group with cccDNA ratio>1 (example like # 161 in Fig 3).

Next, to confirm the predication that PCR products amplified from the cccDNA in the CT group had more AT-rich DNA due to deamination than the CNCT group, DNAs amplified at 84.1°C from matched CT and CNCT samples (#110 and #178) were cloned and sequenced. Sequence analysis of 13 individual clones revealed that more G to A mutations accumulated in CT group than in the CNCT group (Fig 3B). The frequency of G to A changes was 4.85 per 1000 sites in CT group, while it was only 0.16 in the CNCT group. These results demonstrated that G to A mutations accumulated 30-fold more frequently in the CT samples than in matched CNCT samples.

We noted the dinucleotide context for these G to A hypermutations in CT group showed preferred editing in the GpA motifs (66.7%, Fig 3C). This implies the editing may have been performed by one or more of the APOBEC3 proteins, consistent with previous studies that revealed that editing in GpA and GpG of dinucleotide context was a hallmark for APOBEC3 deaminases [18,22].

**Fig 4:**
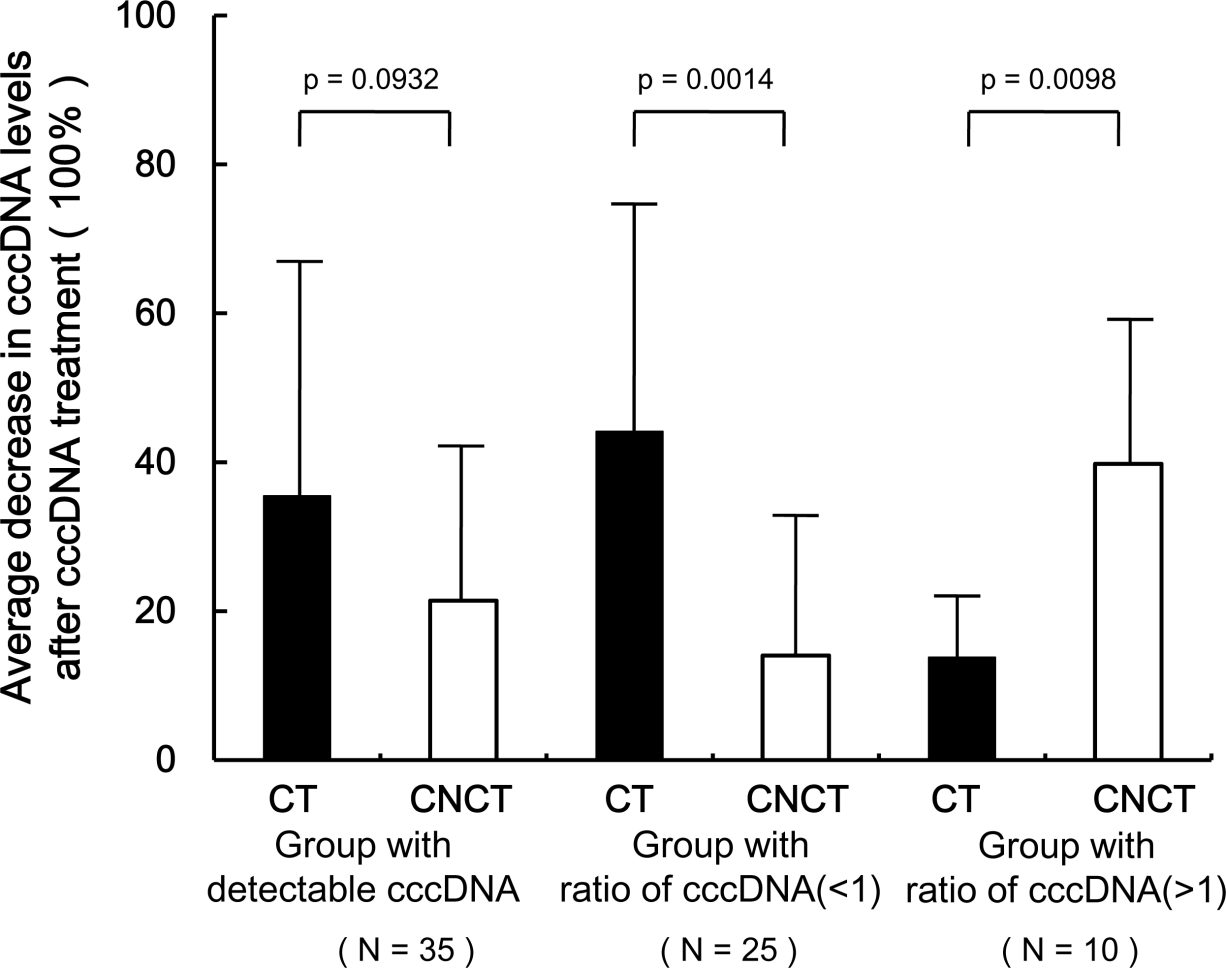
Effect of APE1 treatment on detection of cccDNA levels in 35 paired CT and CNCT samples. The 35 paired HCC samples with detectable cccDNA values (left column, N = 35) were divided into two group according to ratio of cccDNA in the paired CT and CNCT samples (CT/CNCT) < 1 (middle column, N = 25) or >1 (right column, N = 10). Extracted tissue DNAs containing the cccDNA were treated with APE1, and cccDNA levels were measured before and after treatment. The decrease in cccDNA levels in each sample was calculated as = (value before APE1 treatment—value after APE1 treatment) / value before APE1 treatment. CT: cancerous tissues, CNCT: Contiguous noncancerous tissues.

To further investigate whether the cccDNA contain AP sites whose levels may correlate with the loss of cccDNA in the CT samples, we quantified cccDNA levels before and after treatment by APE1 in 35 paired HCC samples because APE1 cleaves the phosphodiester backbone within an AP site. The mean decrease in cccDNA levels in the CT group following AP1 treatment was 1.7-fold greater than that in CNCT group (35.48% vs 21.39%, p = 0.0932, Fig 4), and was 3.1 fold higher in the group of samples where the CT/CNCT cccDNA ratio was < 1 (44.15% vs 14.04%, p = 0.0014).

### Expression levels of APOBEC3 deaminases in HCC liver tissues

**Fig 5:**
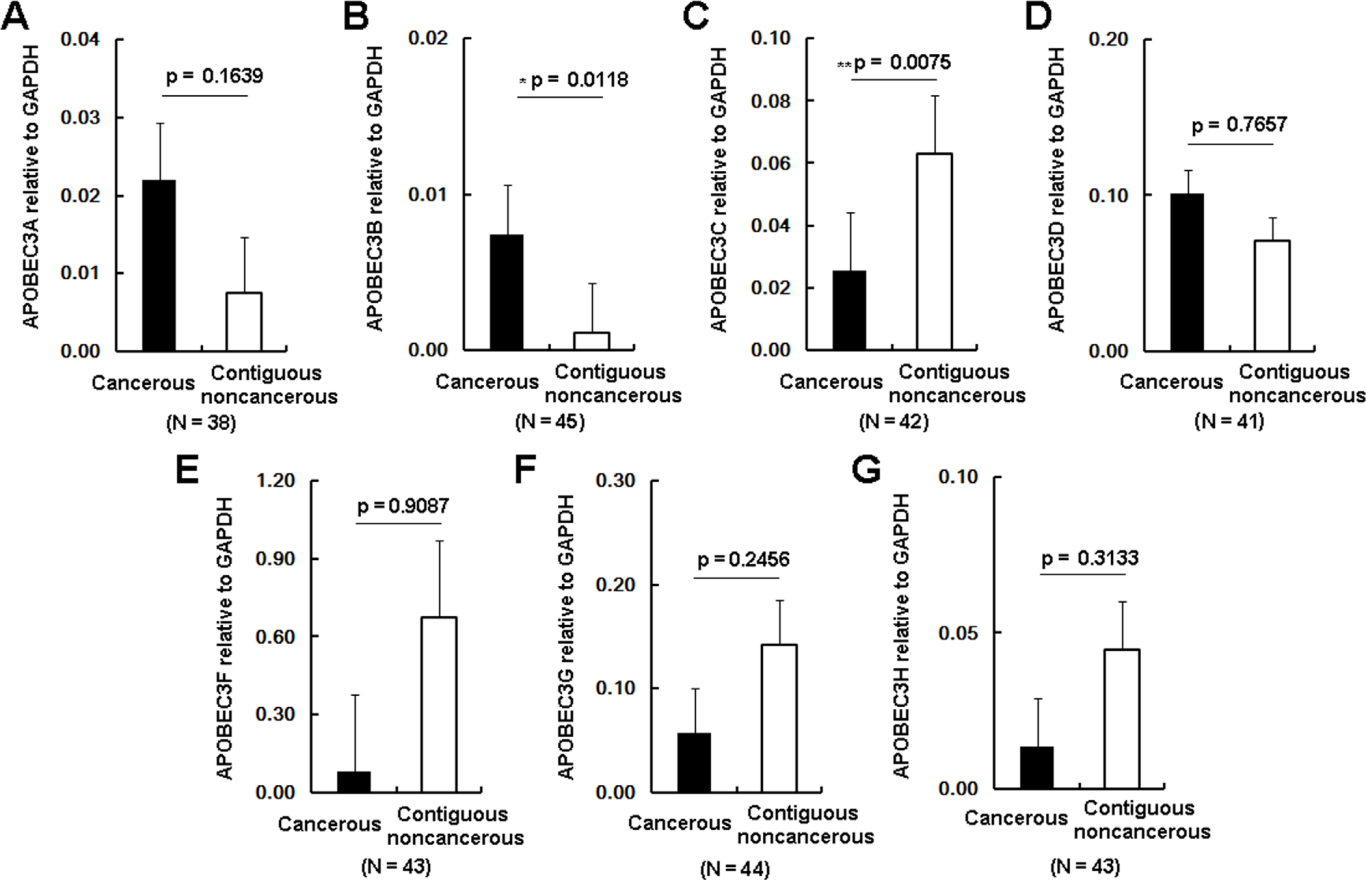
The transcription profile of the 7 APOBEC3 genes in the CT group and matched CNCT group in HCC patients. Levels of the target APOBEC3 mRNAs were measured by qRT-PCR and normalized to the expression levels of GAPDH. N represents the number of HCC patients with detectable expression of the 7 target APOBEC3 mRNAs. Asterisks indicate statistically significant differences: * 0.01< p < 0.05, ** 0.005< p < 0.01.

**Fig 6:**
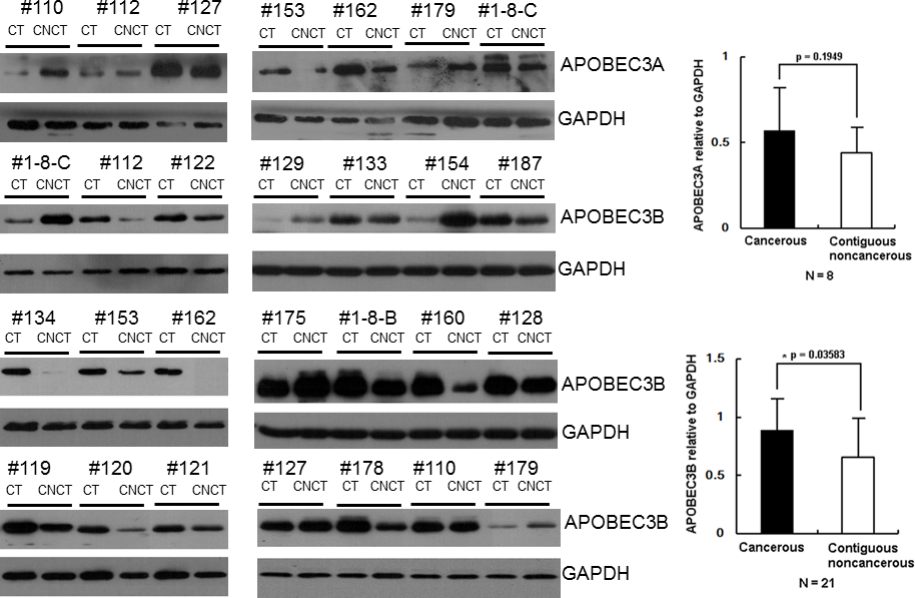
APOBEC3B is widely up-regulated in HCC. **A**. Protein levels of APOBEC3A and APOBEC3B in HCC samples were measured by Western blotting using anti-APOBEC3A and APOBEC3B antibodies; GAPDH was used as control for protein loading. CT: cancerous tissues, CNCT: Contiguous noncancerous tissues. Qualification of protein levels of APOBEC3A (panel **B**) and APOBEC3B (panel **C**) in HCC by normalization to the expression levels of GAPDH.

Next, the transcription profile of the APOBEC3 genes in these 49 HCC samples was measured by RT-qPCR with normalization to GAPDH. As shown in Fig 4, APOBEC3A, APOBEC3B and APOBEC3D were up-regulated in the CT group compared to the CNCT group, while APOBEC3C, APOBEC3E, APOBEC3F and APOBEC3G were down-regulated. Changes in the levels of APOBEC3B and APOBEC3C achieved statistical significance (APOBEC3B, p = 0.01, Fig 5B; APOBEC3C, p = 0.0075, Fig 5C). The proportion of samples in which APOBEC3B was up-regulated was 65% (29/45) in the CT tissues, while the proportion of up-regulated APOBEC3A was 37.5% (15/40). Considering APOBEC3A and APOBEC3B had been reported to be factors in limiting cccDNA levles, we conducted Western blot analysis of APOBEC3A and APOBEC3B from these liver tissues. Western blot from 21 paired HCC samples indicated the levels of APOBEC3B protein in CT samples were also widely up-regulated compared to the matched CNCT tissues (APOBEC3B, p = 0.0358, Fig 6). Again, the levels of APOBEC3A protein were up-regulated in the CT group, but this did not achieve statistical significance (APOBEC3A, p = 0.1942, Fig 6). Combined with the G to A mutations analysis, these data are consistent with up-regulation of APOBEC3B contributing to editing and degradation of the cccDNA in HCC patients.

## Discussion

HCC is one of the most frequent fatal cancer in the world, and it is estimated about 55% HCC are related to chronic infection with HBV [30,31]. Although the exact mechanism of HBV-induced HCC remains unclear, accumulated evidence has shown HBx protein is a multifunctional regulator in the pathogenesis of HCC [32]. In addition, HBV integration into host genome and alters expression of several genes that contribute to transforming hepatocytes and promote HCC development [33]. The essential role of the cccDNA in maintaining HBV infection indicated that it should be central in HCC development, albeit indirectly [34]. This indicates that investigating intrahepatic cccDNA levels of HCC patients is important.

Here, we found that about 71% (35/49) and 96% (47/49) of HCC patients had detectable cccDNA in CT and CNCT tissues, respectively. In the CT group, the levels of cccDNA ranged from 0.00114 copies/cell to 41.02 copies/cell, and this correlated with the amount of total intrahepatic HBV DNA (Fig 2). A similar result was observed by Wang et al [13]. When comparing the levels of cccDNA, the mean intrahepatic cccDNA in the CT group was significantly decreased by 1.8 fold than in the CNCT group (p = 0.0033, Fig 1), and cccDNA levels in the CT group tended to be distributed among the lower values. Previously, Wong et al had measured and compared the cccDNA levels in CT and CNCT samples, and showed the CT group had higher cccDNA levels [34]. The reason for this discrepancy is unknown, but may be due to the following reasons. First, PSAD enzyme was used to digest HBV double-stranded linear DNA and single-stranded DNA prior to quantification of cccDNA levels in our study, which would increase the accuracy for cccDNA qualification [35]. Second, the criterion of sample grouping is different for the two studies. HBsAg-negative patients were included in our research while they were excluded in Wong’s report. Several other studies have reported that in HBsAg-negative patients, cccDNA levels were still detectable and were at lower levels than in HBsAg-positive carriers [10,11]. Finally, our study subjects were all Han Chinese, mostly from Chongqing area with typical HBV genotype B and C infections, but we have no information about patients in Wong et al’s study. Therefore, further large scale population analyses need to be done to confirm this. Another issue is HBV cccDNAs do not replicate [36], and it is not clear how cccDNA is distributed into daughter cells during cell division. Meanwhile, it is difficult to quantify the hepatocyte turnover levels in HBV infected patients [37]. Therefore, our study cannot exclude the possibility that some of the loss of cccDNA may be due to proliferation of cancerous cells.

The observation that cccDNA levels in the tumor tissues decreased prompted us seek factors that may be involved in this process. APOBEC3 proteins have been identified as potent inhibitors of HBV, but the molecular mechanism of APOBEC3 inhibition is not completely clear. The mechanism of inhibition for HBV replication has been reported as being both dependent or independent of APOBEC3 enzymatic activity. Bonvin et al found APOBEC3B inhibition of HBV replication was dependent on hypermutation, with G to A accumulating in the plus-polarity DNA strand during reverse transcription [38]. Another report also found APOBEC3G is encapsidated with pgRNA in the core particles and interferes in the process of minus-strand DNA synthesis [39]. Recently, Lucifora et al found APOBEC3A and APOBEC3B are up-regulated by IFN -α and lymphotoxin-β-receptor, respectively, causing cccDNA degradation. These reports demonstrated the importance of APOBEC3 proteins against HBV replication, however, the mechanism still remains unclear, with a number of questions remaining. First, does APOBEC3B edit the HBV RNA or DNA? Although HBV viral RNA were reduced in the presence of APOBEC3B in HepG2.0 cells [40], our data revealed that APOBEC3B does not edit HBV RNA directly (S3 Fig). Another question that is still a matter of debate is whether APOBEC3 deaminates the single strand DNA during reverse transcription in the cytoplasm and/or if it deaminates double strand cccDNA in the nucleus [41] because the rate of deamination for APOBEC3 is 200 or 300 fold higher on ssDNA than dsDNA *in vitro* [42]. Therefore, further study focusing on how APOBEC3 proteins bind to cccDNA or single-stranded HBV DNA and whether the deamination pattern is similar or different between cccDNA and DNA from core particles would help to clarify this issue.

In our study, we found APOBEC3B was the only APOBEC3 family member significantly up-regulated in HCC patients (Fig 5). Other reports had indicated APOBEC3B was up-regulated in many types of tumor tissues, including ovarian [43], breast and lung carcinomas [44]. Furthermore, as APOBEC3 is mainly located in nucleus [45], which indicates that the reduced cccDNA levels in the CT samples may be in part due to up-regulated APOBEC3B. To clarify this point, we employed RCA and 3D-PCR to analyze the mutations of cccDNA *in vivo*. We found that more G to A mutations accumulated in the CT group than that in CNCT group (Fig 3), in accordance with the lower levels of cccDNA in the CT group. In addition, dinucleotide context analysis showed GpA motifs were the dominating editing profile for these G to A hypermutations in CT group. These results are consistent with APOBEC3B-mediated deamination contributing to the decline in cccDNA in HCC tissues. Meanwhile, it is reasonable to point out APOBEC3A may also contribute to this effect, but it likely plays a lesser role as it up-regulation did not achieved statistical significance (Fig 6).

In summary, our findings demonstrate that cccDNA levels are significantly lower in HCC tissues, and that the lower levels are likely to stem in part from up-regulation of APOBEC3B, which would target the HBV cccDNA and cause its deamination-dependent degradation. These results provide support for the potential efficacy of inducing APOBEC3B activity as part of an anti-HBV therapeutic regimen.

## Supporting Information

S1 Fig Specificity of Rolling Circle Amplification for cccDNA. **A**. Specificity of RCA to amplify cccDNA in cell culture or liver tissues. cccDNA purified from HepAD38 or clinical patients was amplified by RCA; DNA from core particles and genomic DNA from HBV- transgenic mice were used as controls. **B**. RCA product from line 2, line3 and line 4 in Fig A amplified by genomic PCR. C. Southern blot of RCA product amplified by genomic PCR.
(TIF)

S2 Fig 3D-PCR of 35 paired HCC samples. CT and CNCT samples from the HCC patients were divided into 2 groups with ratio of cccDNA in the paired samples (CT/CNCT) being either < 1 or > 1. cccDNA was specifically amplified by RCA, then the concatemerized product was analysis by 3D-PCR using a denaturation temperature gradient of 86°C-82°C.
(TIF)

S3 Fig APOBEC3B does not edit HBV RNA. **A**. Southern blot of HBV core-associated DNA with transfected with a replication-incompetent HBV genomic expression vector (YMHA plasmid). **B**. 3D-PCR of cDNA reverscribed from RNA in lysates from cells transfected with the YMHA plasmid. Huh7.0 cells were cotransfected with YMHA plasmid plus empty plasmid or APOBEC3B expression plasmid. Total RNA was purified and digested with DNase I, then RT-PCR products served as templates for analysis by 3D-PCR with denaturing temperature ranging from 82–90°C. **C.** Mutation matrices of RT-PCR products. DNA fragments amplified at 94°C in the presence of empty vector and APOBEC3B were cloned into T vectors, and 15 clones were randomly selected and sequenced.
(TIF)

S1 Table Sequences of primers and probe. (DOC)

S2 Table Shapiro-Wilk Normality test. (DOC)

## Abbreviations

HBV: hepatitis B virus
cccDNA: covalently-closed circular DNA
RC DNA: relaxed circular DNA
pgRNA: pregenomic RNA
HBsAg: hepatitis B surface antigen
HBcAb: hepatitis B core antibody
HBeAg: hepatitis B e antigen
HCC: hepatocellular carcinoma
CT: cancerous tissues
CNCT: contiguous noncancerous tissues
APOBEC3: apolipoprotein B mRNA-editing catalytic polypeptide 3
RCA: rolling circle amplification
3D-PCR: differential DNA denaturation PCR
UNG: uracil-DNA glycosylase
