# Supplementary figures and images for: Association of Hepatitis B Virus Covalently Closed Circular DNA and Human APOBEC3B in Hepatitis B Virus-Related Hepatocellular Carcinoma
Source: PLoS One. 2016 Jun 16;11(6):e0157708. doi: 10.1371/journal.pone.0157708 (PMC4911053; doi:10.1371/journal.pone.0157708)

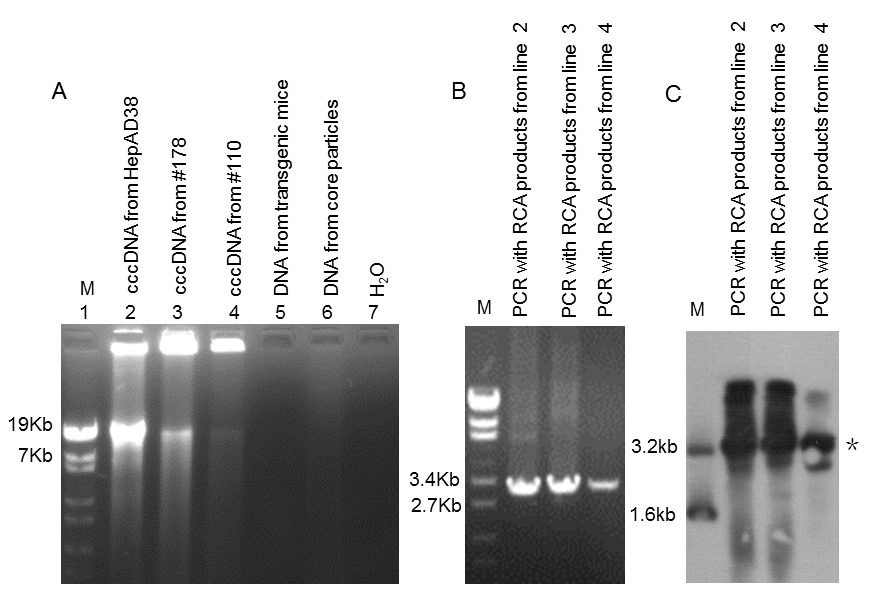

Supplement: S1 Fig — A. Specificity of RCA to amplify cccDNA in cell culture or liver tissues. cccDNA purified from HepAD38 or clinical patients was amplified by RCA; DNA from core particles and genomic DNA from HBV- transgenic mice were used as controls. B. RCA product from line 2, line3 and line 4 in Fig A amplified by genomic PCR. C. Southern blot of RCA product amplified by genomic PCR. (TIF) [file pone.0157708.s001.tif]

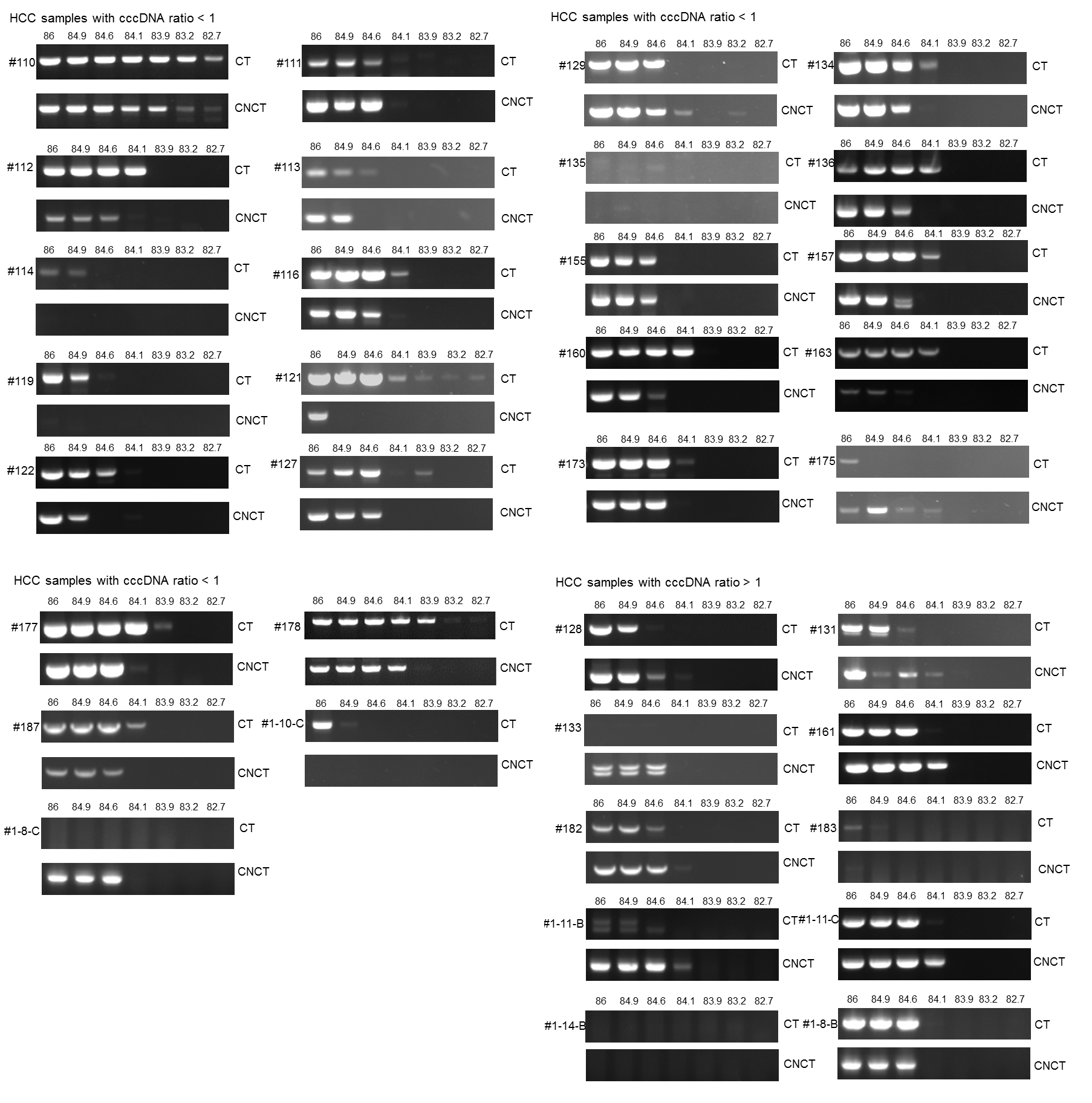

Supplement: S2 Fig — CT and CNCT samples from the HCC patients were divided into 2 groups with ratio of cccDNA in the paired samples (CT/CNCT) being either < 1 or > 1. cccDNA was specifically amplified by RCA, then the concatemerized product was analysis by 3D-PCR using a denaturation temperature gradient of 86°C-82°C. (TIF) [file pone.0157708.s002.tif]

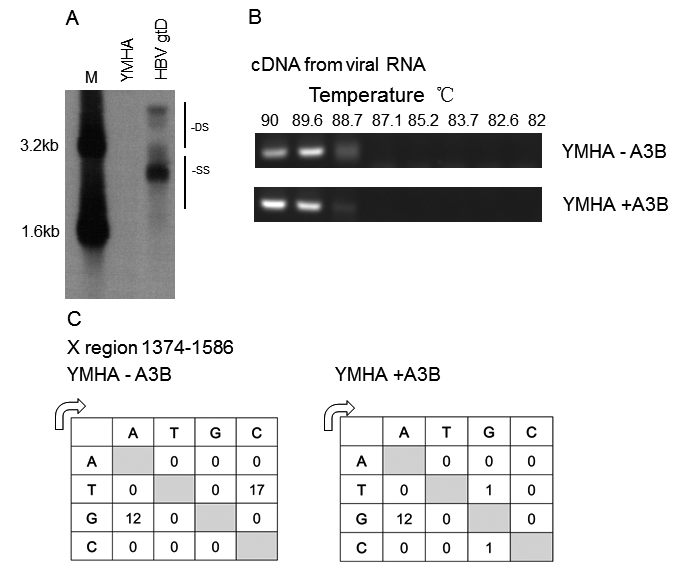

Supplement: S3 Fig — A. Southern blot of HBV core-associated DNA with transfected with a replication-incompetent HBV genomic expression vector (YMHA plasmid). B. 3D-PCR of cDNA reverscribed from RNA in lysates from cells transfected with the YMHA plasmid. Huh7.0 cells were cotransfected with YMHA plasmid plus empty plasmid or APOBEC3B expression plasmid. Total RNA was purified and digested with DNase I, then RT-PCR products served as templates for analysis by 3D-PCR with denaturing temperature ranging from 82–90°C. C. Mutation matrices of RT-PCR products. DNA fragments amplified at 94°C in the presence of empty vector and APOBEC3B were cloned into T vectors, and 15 clones were randomly selected and sequenced. (TIF) [file pone.0157708.s003.tif]
