# Supplementary material for: Association of Hepatitis B Virus Covalently Closed Circular DNA and Human APOBEC3B in Hepatitis B Virus-Related Hepatocellular Carcinoma
Source: PLoS One. 2016 Jun 16;11(6):e0157708. doi: 10.1371/journal.pone.0157708 (PMC4911053; doi:10.1371/journal.pone.0157708)
Supplement: S1 Table — (DOC) [file pone.0157708.s004.doc]

**S1 Table. Sequences of primers and probe**

| **Prime and probe** | **Sequence (5’to 3’)** | **Position** |
| --- | --- | --- |
| Prime and probe for HBV cccDNA * |  |  |
| cccDNAFP | CTCCCCGTCTGTGCCTTCT | 1548-1566 |
| cccDNARP | CCCCAAAGCCACCCAAG | 1903-1886 |
| cccDNAprobe | FAM- ACGTCGCATGGAGACCACCGTGAACGCC- TAMRA | 1603 |
| Prime and probe for HBV total DNA |  |  |
| HBVDNAFP | TGCGGCGTTTTATCATATTCC | 384-395 |
| HBVDNARP | ATACCTTGGTAGTCCAGAAGAACCA | 438-414 |
| cccDNAprobe | FAM-TTCATCCTGCTGCTATGCCTCATCTTCTTG - TAMRA | 407-437 |
| Prime and probe for human globin |  |  |
| GlobinFP | ACCCAGAGGTTCTTTGAGTCCTT | 1867-1890 |
| GlobinRP | GCCATGAGCCTTCACCTTAGG | 1947-1927 |
| Globinprobe | FAM- TCCACTCCTGATGCTGTTATGGGCAA- TAMRA | 1888-1914 |
| Prime of full-length genomic PCR ** |  |  |
| P1 | ccggaaagcttgACTTTTTCACCTCTGCCTAATC | 1819-1840 |
| P2 | ccggaaagcttgtcAAAAAGTTGCATGGTGCTGG | 1823-1804 |
| Prime of 3D-PCR of Core region |  |  |
| 3D-FP core | CCGCCTCAGCTCTGTATC | 1998-2015 |
| 3D-RP core | TCCCACCTTATGAGTCCA | 2460-2477 |
| Prime of 3D-PCR of X region*** |  |  |
| 3D-OFP X | CGCAAATATACATCGTATCCAT | 1354-1375 |
| 3D-ORP X | AAGAGTYYTYTTATGTAAGACYTT | 1644-1667 |
| 3D-IFP X | ATGGCTGCTARGCTGTGCTGCCAA | 1374-1397 |
| 3D-IRP X | AAGTGCACACGGTYYGGCAGAT | 1565-1586 |
| Prime of APOBEC3 **** |  |  |
| APOBEC3AFP | GAGAAGGGACAAGCACATGG |  |
| APOBEC3ARP | TGGATCCATCAAGTGTCTGG |  |
| APOBEC3BFP | GACCCTTTGGTCCTTCGAC |  |
| APOBEC3BRP | GCACAGCCCCAGGAGAAG- |  |
| APOBEC3CFP | AGCGCTTCAGAAAAGAGTGG |  |
| APOBEC3CRP | AAGTTTCGTTCCGATCGTTG |  |
| APOBEC3DFP | ACCCAAACGTCAGTCGAATC |  |
| APOBEC3DRP | CACATTTCTGCGTGGTTCTC |  |
| APOBEC3FFP | CCGTTTGGACGCAAAGAT |  |
| APOBEC3FRP | CCAGGTGATCTGGAAACACTT |  |
| APOBEC3GFP | CCGAGGACCCGAAGGTTAC |  |
| APOBEC3GRP | TCCAACAGTGCTGAAATTCG |  |
| APOBEC3HFP | AGCTGTGGCCAGAAGCAC |  |
| APOBEC3HRP | CGGAATGTTTCGGCTGTT |  |
| GAPDHFP | GAAGGTGAAGGTCGGAGTC |  |
| GAPDHRP | GAAGATGGTGATGGGATTTC |  |
|  |  |  |

*Primers for qPCR amplification of HBV cccDNA were obtained from Werle et al [[1](#_ENREF_1)].

** Primers for HBV full-length genomic PCR were obtained from Margeridon et al [[2](#_ENREF_2)].

*** Primers for 3D-PCR amplified X region of HBV were obtained from Suspene, et al [[3](#_ENREF_3)]

****Primers for quantitation of APOBEC3 transcription levels were obtained from Ohba et al [[4](#_ENREF_4)].

**References**

1. Werle–Lapostolle B, Bowden S, Locarnini S, Wursthorn K, Petersen J, et al. (2004) Persistence of cccDNA during the natural history of chronic hepatitis B and decline during adefovir dipivoxil therapy. Gastroenterology 126: 1750-1758.

2. Margeridon S, Carrouee-Durantel S, Chemin I, Barraud L, Zoulim F, et al. (2008) Rolling circle amplification, a powerful tool for genetic and functional studies of complete hepatitis B virus genomes from low-level infections and for directly probing covalently closed circular DNA. Antimicrob Agents Chemother 52: 3068-3073.

3. Suspene R, Guetard D, Henry M, Sommer P, Wain-Hobson S, et al. (2005) Extensive editing of both hepatitis B virus DNA strands by APOBEC3 cytidine deaminases in vitro and in vivo. Proc Natl Acad Sci U S A 102: 8321-8326.

4. Ohba K, Ichiyama K, Yajima M, Gemma N, Nikaido M, et al. (2014) In vivo and in vitro studies suggest a possible involvement of HPV infection in the early stage of breast carcinogenesis via APOBEC3B induction. PLoS One 9: e97787.
