# Supplementary material for: Association of Hepatitis B Virus Covalently Closed Circular DNA and Human APOBEC3B in Hepatitis B Virus-Related Hepatocellular Carcinoma
Source: PLoS One. 2016 Jun 16;11(6):e0157708. doi: 10.1371/journal.pone.0157708 (PMC4911053; doi:10.1371/journal.pone.0157708)
Supplement: S2 Table — (DOC) [file pone.0157708.s005.doc]

**S2 Table. Shapiro-Wilk Normality test**

| **Normality test（Shapiro-Wilk）** | **cccDNA (copies/ul)**  **N = 35** | | **Total DNA (copies/ul)**  **N = 35** | | **Serum DNA**  **(log copies/ml)**  **N = 26** |
| --- | --- | --- | --- | --- | --- |
|  | Cancerous | Contiguous noncancerous | Cancerous | Contiguous noncancerous |  |
| W | 0.8963 | 0.8546 | 0.8641 | 0.8495 | 0.6464 |
| P | 0.00315 | 0.0002934 | 0.0004898 | 0.000225 | 0.0075 |
